# Double‐bowtie filter design for pediatric spectral CT imaging: A fan beam simulation study

**DOI:** 10.1002/mp.70752

**Published:** 2026-11-05

**Authors:** Yinglin Ge, Olivia F. Sandvold, Roland Proksa, Amy E. Perkins, Thomas Koehler, Kevin M. Brown, Yannan Jin, Heiner Daerr, Ravindra M. Manjeshwar, Peter B. Noël

**Affiliations:** ^1^ Department of Radiology Perelman School of Medicine University of Pennsylvania Philadelphia Pennsylvania USA; ^2^ Department of Bioengineering University of Pennsylvania Philadelphia Pennsylvania USA; ^3^ Philips Healthcare Cleveland Ohio USA; ^4^ Philips Innovative Technologies Hamburg Germany

**Keywords:** bowtie filter, fan beam simulation, K‐edge material, kVp‐switching CT, pediatric imaging, spectral CT

## Abstract

**Background:**

Despite concerns about radiation dose in pediatric patients, computed tomography (CT) remains a first‐line imaging modality for children. Spectral CT provides additional diagnostic information that benefits pediatric imaging. However, most existing spectral CT systems are primarily optimized for adult patients.

**Purpose:**

To develop and evaluate a novel double bowtie filter integrating a K‐edge material layer with a conventional Teflon filter for CT. The proposed design aims to enhance spectral signal‐to‐noise ratio (SNR) and spectral separation within the projection domain while maintaining radiation dose levels suitable for pediatric imaging.

**Methods:**

A simulation framework was developed and used to model a rapid kVp‐switching CT system operating at 70/110 kVp with realistic tube power and geometric constraints. Pediatric phantoms of three sizes (100–200 mm anterior–posterior width) were used to evaluate performance. Five accessible and safe filter materials—gadolinium (Gd), holmium (Ho), erbium (Er), silver (Ag), and tin (Sn)—were tested in combination with a Teflon bowtie. System performance was quantified using virtual monoenergetic image (VMI) SNR at 40 and 70 keV, and the area under the monoenergetic SNR curve (AUMC) as a comprehensive spectral image quality metric. Dose consistency was ensured using a traditional Teflon bowtie as the reference.

**Results:**

The Teflon + Gd configuration achieved the highest performance, improving mean AUMC over all fan angles by 53.6% on average and up to 56% for the largest phantom. VMI SNR increased by approximately 46% at 40 keV and 42% at 70 keV.

**Conclusions:**

The double‐bowtie concept substantially enhances spectral performance. The Teflon + Gd design provides a manufacturable, pediatric‐optimized solution adaptable to kVp‐switching and other spectral CT architectures, offering improved diagnostic quality at low dose levels.

## 1 INTRODUCTION

Unlike conventional CT, which merges all X‐ray energies into a single signal, spectral CT distinguishes between different energy levels, allowing it to differentiate materials that appear similar on conventional scans.^1^, ^2^ Spectral data enables the creation of material maps and provides access to quantitative biomarkers,^3^ supporting more accurate diagnostic screening, disease staging, and treatment monitoring. Spectral CT also enables the reconstruction of virtual monoenergetic images (VMI), which can enhance iodine contrast visibility, improve attenuation consistency, and reduce artifacts.^2^, ^4^, ^5^, ^6^ These capabilities offer significant clinical advantages across a wide range of applications. While minimizing radiation exposure remains paramount in pediatric imaging, CT continues to be the gold standard for children because of its high speed and superior spatial resolution. Spectral CT has demonstrated clinical advantages in pediatric diagnostics compared to conventional CT, including improved lesion characterization and artifact reduction.^7^

There are several spectral CT instrumentations, including source‐based and detector‐based techniques. Source‐based systems contain dual‐source dual‐energy, single‐source kilovoltage switching (kVp‐switching),^8^ and split‐beam systems such as twin‐beam dual‐energy,^9^ which use additional filtration.^10^, ^11^ Detector‐based spectral CT includes dual‐layer and photon‐counting,^12^, ^13^, ^14^ each offering distinct performance in energy resolution and spectral separation.^15^ In spectral CT, spectral separation refers to the system's ability to distinguish between low‐ and high‐energy X‐ray photons. This separation is essential for accurate and stable material decomposition and contrast agent quantification. By increasing spectral separation, the reliability and precision of measurements are improved, resulting in better image quality for all results and the possibility of a lower radiation dose.^16^ Low tube voltages are commonly used in pediatric CT protocols to reduce radiation dose.^17^ However, this results in reduced spectral separation in spectral CT.^18^, ^19^

K‐edge materials, which exhibit sharp increases in X‐ray absorption at specific photon energies, offer a potential solution. By selectively attenuating parts of the spectrum, they can enhance spectral separation (Figure 1). Incorporating a K‐edge filter between the X‐ray source and the patient generates a spectrally shaped beam, improving energy discrimination and potentially restoring or improving image quality even at lower tube voltages.^20^, ^21^

**FIGURE 1:**
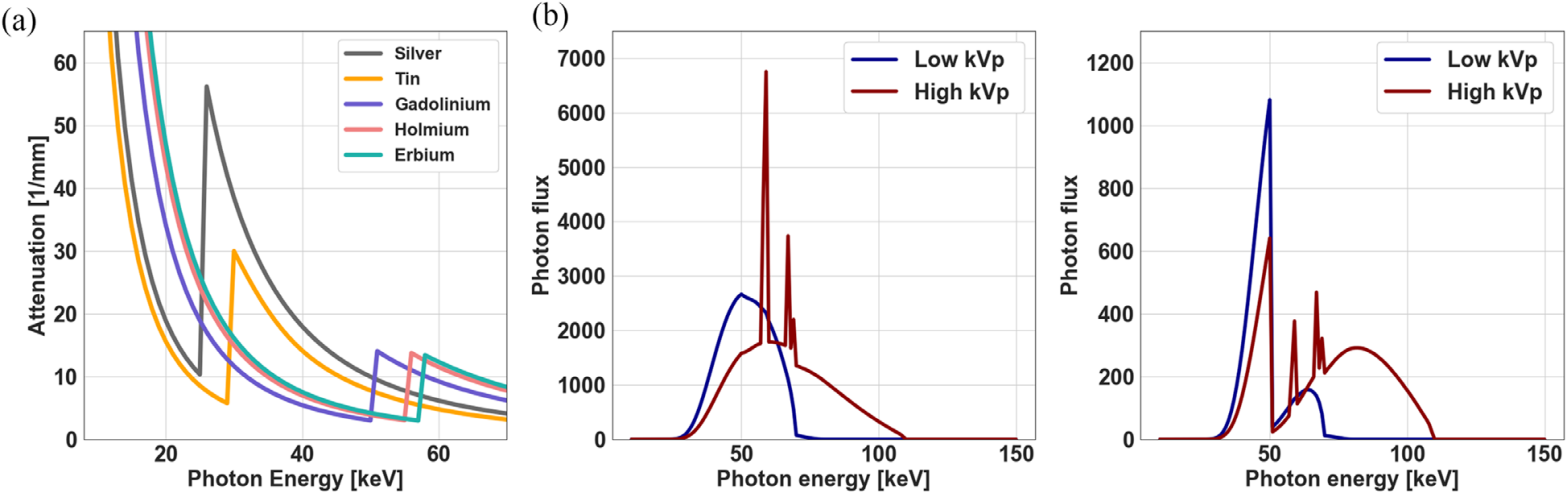
Attenuation of K‐edge materials and spectra before and after K‐edge filtration. (a) Attenuation curve of selected K‐edge materials. (b) *Left*: Low kVp (70 kVp) and high kVp (110 kVp) spectra of simulated kVp‐switching systems. *Right*: Spectra attenuated by 0.3 mm gadolinium.

In CT technology, the bowtie filter remains a critical hardware component. Its fan‐angle‐dependent shape is designed to reduce unnecessary radiation to the patient's periphery, helping to optimize dose distribution. Additionally, bowtie filters help equalize the X‐ray signal across the detector array, reducing pulse pile‐up effects for photon‐counting detectors (PCDs).^22^ Teflon (CF_2_) is widely used as a bowtie filter material due to its attenuation properties, which closely resemble those of water, making it approximately tissue‐equivalent.^23^ However, thicker Teflon filters can contribute to increased scatter, and geometrical constraints of the CT gantry may limit the feasible thickness. Beyond material choice, bowtie filters can be tailored for specific anatomical regions to compensate for variations in tissue composition and shape.^24^, ^25^ Non‐traditional designs, such as dynamic bowtie filters,^26^, ^27^, ^28^, ^29^ further aim to adapt the filter shape across different projection angles for improved patient matching. However, these approaches focus largely on optimizing material choice and geometry, with little integration of spectral CT requirements.^30^ Although commercial scanners offer pediatric or small‐body bowtie options,^31^ these are typically scaled‐down versions of the adult bowtie geometry and have not been jointly optimized with spectral CT requirements for the pediatric population, the group most vulnerable to radiation exposure.

Although CT is widely used in pediatric imaging, most commercial CT systems are optimized for adults.^32^ To address this challenge, we propose a novel double bowtie prefiltration design that pairs a conventional Teflon bowtie filter with a K‐edge filter, delivering enhanced image quality for pediatric spectral CT imaging. The K‐edge material selectively shapes the X‐ray spectrum, enhancing spectral separation, while its strong attenuation properties allow for a thinner Teflon bowtie, thereby reducing scatter. Meanwhile, the bowtie filter continues to fulfill its essential roles in normalizing detector signals and decreasing unnecessary patient dose. In our previous work, this combined design demonstrated improved spectral performance in simulations using a kVp‐switching CT system under parallel beam geometry^33^ for a limited number of K‐edge materials.^34^ In this study, we expand upon that foundation by exploring additional K‐edge materials and optimizing the double bowtie design in fan‐beam geometry for a kVp‐switching CT system. We hypothesize that integrating a K‐edge layer within a conventional bowtie filter can enhance spectral separation and SNR without increasing radiation dose.

## 2 METHODS

### 2.1 System simulation

A Python simulation was designed to model a CT system comprised of clinical‐grade components. X‐ray beamlets were modeled from a prototype rapid kVp‐switching tube (vMRC 800, Philips Healthcare) with a maximum tube power limit of 80 kW. For kVp‐switching configurations, voltage transitions were governed by a fixed generator ramp rate, so that the rise time scaled with the magnitude of the voltage difference and the fall time scaled with both the voltage difference and the tube current. The X‐ray spectra emitted from the anode was generated using spekCalc software^35^ with photon energies from 10 to 150 keV. The spectra after filtration and the realistic detector responses were then simulated (Figure 2). The material and thickness of the inherent filtration of the X‐ray tube were incorporated into the model. Photon scattering and absorption data of materials were sourced from the National Institute of Standards and Technology (NIST) XCOM dataset.^36^ The CT gantry geometry was also modeled based on the clinically feasible CT system.

**FIGURE 2:**
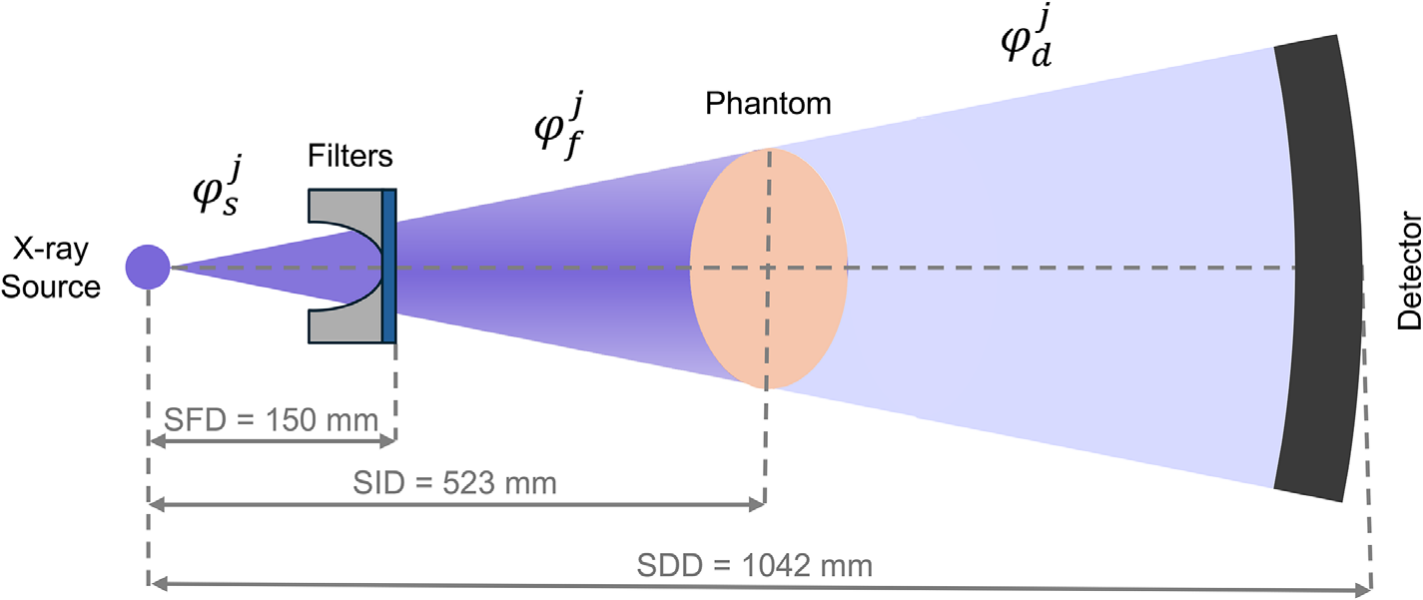
System sketch. For each ray j, the initial X‐ray spectrum was denoted as $\varphi_{s}^{j}$, the spectrum after passing the bowtie filter was denoted as $\varphi_{f}^{j}$, and the spectrum after passing the phantom and collected by the detector was denoted as $\varphi_{d}^{j}$. The cosine effect was applied to all j th X‐rays outside of 0 degrees in the simulated fan‐beam. The source‐to‐filter distance (SFD) was 150 mm, the source‐to‐isocenter distance (SID) was 523 mm, and the source‐to‐detector distance (SDD) was 1042 mm.

The tube voltage waveform of the kVp‐switching system is shown in Figure 3. The X‐ray spectra corresponding to the kV waveform with proper transition was simulated, and the tube current was set properly for each voltage. Higher tube current (higher tube output) shortens the voltage transition duration during ramp‐down, which reduces spectral overlap. To capture the spectral content during voltage transitions, the tube voltage waveform was sampled at high temporal resolution, and the instantaneous spectra, including those during the rise and fall ramps, were integrated over each integration period to form its effective spectrum, with the ramp contribution assigned to the high‐ or low‐kVp channel at the threshold voltage. In this study, X‐ray exposure was simulated at tube voltage pairs of 110 kVp (high‐kVp, $\varphi_{110}$) and 70 kVp (low‐kVp, $\varphi_{70}$) with an average tube current of 100 mA. Under these settings, the rise time was approximately 29.3 µs and the fall time approximately 114.6 µs. The total rotation time was 1000 ms, with a duty cycle of 10/90, indicating the partition of the plateau time between the high‐ and low‐kVp levels; the effective high‐ and low‐kVp integration periods, split at the threshold voltage, were approximately 177 and 656 µs per 833 µs cycle (Figure 3). The 10/90 duty cycle was selected as a balance among dose, spectral performance, and hardware feasibility.^37^ A total of 2400 readings (1200 for each voltage) were acquired per rotation. These settings defined the reference system, mimicking a dose level comparable to standard pediatric abdominal or pediatric chest imaging protocols.

**FIGURE 3:**
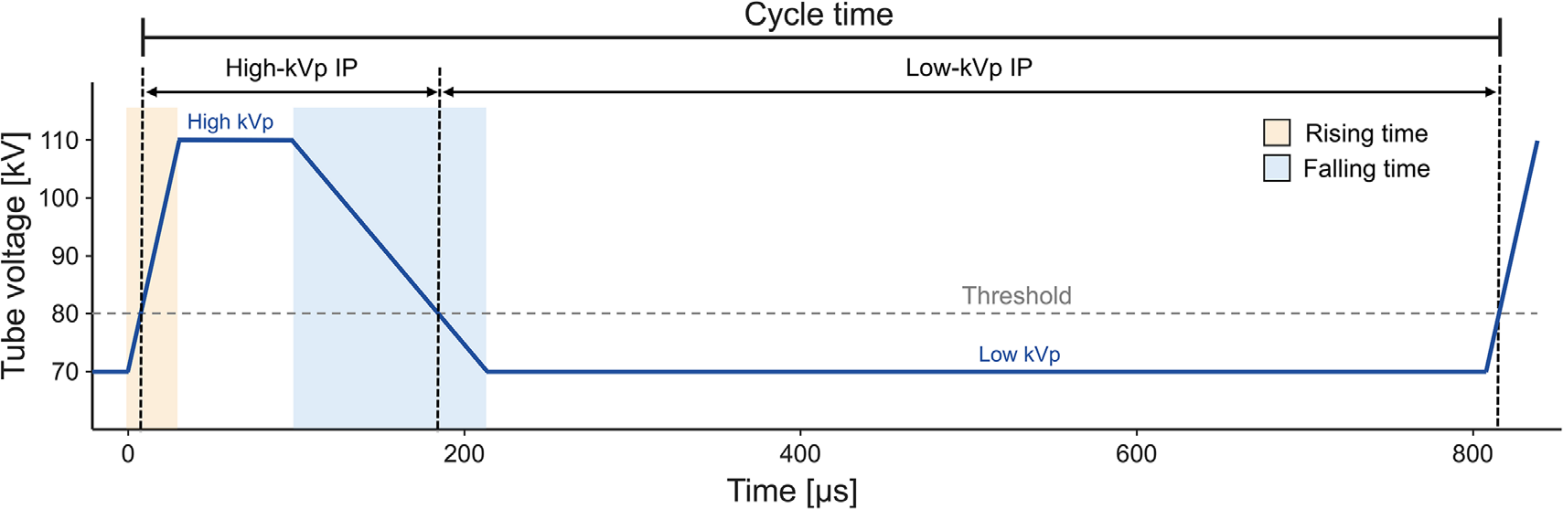
Tube voltage waveform of the kVp‐switching system for the 110/70 kVp reference protocol (100 mA, 10/90 duty cycle). One cycle (833 µs) spans between successive threshold crossings of the rising ramp. The rise (29.3 µs) and fall (114.6 µs) transitions, determined by the voltage difference and the tube current, are shaded. The duty cycle partitions the remaining plateau time between the two voltage levels. The high‐ and low‐kVp integration periods (IPs), delimited by the threshold voltage (80 kV, at 25% of the voltage difference above the low kVp, dashed line), include the ramp portions above and below it (approximately 177 and 656 µs) and define the high‐ and low‐kVp spectra, respectively.

### 2.2 Phantom design

To model pediatric patients of varying sizes, three elliptical water phantoms were simulated. The selected phantom sizes represented a broad range of pediatric age groups, with anteroposterior widths of 100 mm, 150 mm, and 200 mm, and transverse widths of 150 mm, 232 mm, and 313 mm. The size corresponded to the average abdominal dimensions of newborns, 8.7‐year‐olds, and 17.5‐year‐olds, respectively.^38^

### 2.3 System evaluation

#### 2.3.1 Detector SNR

The signal‐to‐noise ratio (SNR) of the detector signal was calculated from $\varphi_{d}\left(V,E\right)$ received at the detector, where we assumed the photon shot noise followed the Poisson distribution. In an energy‐integrating detector, each detected photon contributes an amount of signal response proportional to its energy. The signal from the impinging spectrum $\varphi_{110,70}$ can be represented by the total photon energy. The noise is the standard deviation in the distribution, so the detector SNR can be written as

(1) $${\begin{array}{c} SNR \end{array}}_{detector}\;\left(V\right)=\frac{\int_{E=0}^{\infty }\varphi_{d}\left(V,E\right)\cdot E\;dE}{\sqrt{\int_{E=0}^{\infty }\varphi_{d}\left(V,E\right)\cdot E^{2}\;dE\;}}\;$$

where V is the tube voltage. In our simulation, energies from 20 to 150 keV are calculated.

#### 2.3.2 VMI SNR

In this simulation, the Cramér‐Rao lower bound of variance (CRLB) was used to estimate the noise on the photoelectric effect and Compton scatter two‐basis decomposition.^39^ The attenuation coefficient of a material was assumed to be the linear combination of these two basis functions. Spectral CT reconstruction was based on the estimation of line integrals $A_{k}$ along a ray C from two spectra measurements,^40^ where k represents photoelectric effect (pe) or Compton scatter (cs). $a_{k}$ is the spatial density map, often referred to as the basis coefficient.

(2) $$A_{k}=\int_{C}a_{k}\left(x,y\right)ds\;$$

The energy‐dependent attenuation related to a given basis function can be written as

(3) $$p_{k}\;\left(E\right)=A_{k}\;\cdot f_{k}\left(E\right)$$

where $f_{k}$ is the energy‐dependent basis function of photoelectric effect (pe) or Compton scatter (cs). The two spectra incident on the phantom, $\varphi_{f}\left(110\mathrm{kV},E\right)$ and $\varphi_{f}\left(70\mathrm{kV},E\right)$, each represent the effective spectrum integrated over the corresponding integration period, including the voltage transition contribution. The two measurements are assumed to be independent. Each measurement $M_{j}$ was modeled as a Gaussian random variable representing the total energy deposited in the $j^{\mathrm{th}}$ acquisition, with $m_{j}$ denoting its observed value, for j=1,…,N. Let $\theta_{j}$ and $\sigma_{j}^{2}$ be the corresponding mean and variance of the Gaussian distribution measured at tube voltage $V_{j}$ (Equations (4), (5), where the material line integrals receive index j).

(4) $$\theta_{j}=\int E\cdot \varphi_{f}\left(V_{j},E\right)\cdot \exp\left(-\sum_{k\in \left\{\mathrm{pe},\mathrm{cs}\right\}}A_{k,j}\cdot f_{k}\left(E\right)\right)\;\mathrm{dE}$$

(5) $$\sigma_{j}^{2}=\int E^{2}\cdot \varphi_{f}\left(V_{j},E\right)\cdot \exp\left(-\sum_{k\in \left\{\mathrm{pe},\mathrm{cs}\right\}}A_{k,j}\cdot f_{k}\left(E\right)\right)\;\mathrm{dE}$$

Then the probability density $\rho (m_{1},\text{…},m_{N})$ of $M_{j}$ can be calculated as follows:

(6) $$\begin{array}{cc} dP\;\left(m_{1},\cdots ,\;m_{N}\right) & =\rho \left(m_{1},\cdots ,\;m_{N}\right)\prod_{j=1}^{N}dm_{j}\\ & =\prod_{j=1}^{N}\frac{1}{\sqrt{2\pi \sigma_{j}^{2}}}\;e^{-\frac{{\left(m_{j}-\theta_{j}\right)}^{2}}{2\sigma_{j}^{2}}}dm_{j\;} \end{array}$$

The negative log‐likelihood can therefore be written as:

(7) $$\begin{array}{cc} L & =-\ln\rho \;\left(m_{1},\text{…},\;m_{N}\right)\\ & =\sum_{j=1}^{N}\left[\frac{1}{2}ln\;\sigma_{j}^{2}+\frac{{\left(m_{j}-\theta_{j}\right)}^{2}}{2\sigma_{j}^{2}}\right]+\frac{1}{2}\;N\;\ln\left(2\pi \right) \end{array}$$

Because $\theta_{j}$ and $\sigma_{j}^{2}$ depend on the basis line integrals $A_{pe,j}$ and $A_{cs,j}$(Equations (4), (5)), the negative log‐likelihood L is a function of these parameters. The Fisher information matrix, F(θ), can be derived by taking the expectation of the second partial derivative of this function with respect to $A_{pe}$ and $A_{cs},$

(8) $$\begin{array}{cc} F_{k_{1},k_{2}}\left(\theta \right) & =E\;\left[\frac{\partial^{2}L}{\partial A_{k_{1}}\;\partial A_{k_{2}}}\right]\\ & =\sum_{j=1}^{N}\left(\frac{1}{\sigma_{j}^{2}}\frac{\partial \theta_{j}}{\partial A_{k_{1}}}\;\frac{\partial \theta_{j}}{\partial A_{k_{2}}}+\frac{1}{2\sigma_{j}^{4}}\frac{\partial \sigma_{j}^{2}}{\partial A_{k_{1}}}\frac{\partial \sigma_{j}^{2}}{\partial A_{k_{2}}}\right) \end{array}$$

We can now obtain the Cramér–Rao inequality^41^ for the variance of any unbiased estimator of the parameters $A_{pe}$ and $A_{cs}$:

(9) $$F=\left[\begin{array}{cc} F_{pe,pe}\left(\theta \right) & F_{pe,cs}\left(\theta \right)\\ F_{cs,pe}\left(\theta \right) & F_{cs,cs}\left(\theta \right) \end{array}\right]\;$$

where the determinant of the F is:

(10) $$\det\left(F\right)=F_{pe,pe}\;\cdot F_{cs,cs}-F_{pe,cs}^{2}$$

Then the separate CRLB bound for each basis can be written as:

(11) $$\mathrm{Var}\left(A_{pe}\right)\;\geq \;F_{1,1}^{-1}=\frac{F_{cs,cs}}{\det\left(F\right)}\;,\;\mathrm{Var}\left(A_{cs}\right)\;\geq \;F_{2,2}^{-1}=\frac{F_{pe,pe}}{\det\left(F\right)}\;$$

To assess the performance of material decomposition at specific energies, we compute the local SNR at a monoenergetic energy E based on the propagated uncertainty from the CRLB. The variance of each energy level, denoted as $\sigma_{MonoE}^{2}\left(E\right)$, can be calculated from the CRLB covariance matrix [F] ^−1^:

(12) $$\begin{array}{cc} \sigma_{MonoE}^{2}\;\left(E\right) & =f_{pe}^{2}\;\left(E\right)\cdot \mathrm{co}v_{pe,pe}\\ & +2f_{pe}\left(E\right)f_{cs}\left(E\right)\cdot \mathrm{co}v_{pe,cs}+f_{cs}^{2}\left(E\right)\cdot \mathrm{co}v_{cs,cs} \end{array}$$

The estimated noise at each VMI level could then be defined as the square root of the variance, ${\left(\sigma_{\mathrm{MonoE}}^{2}\left(E\right)\right)}^{1/2}.$

In spectral CT, 40 keV VMI is commonly used for enhancing the contrast of iodine contrast agents and soft tissue.^42^, ^43^ Additionally, 70 keV VMI is a better representative of conventional image^44^ and yields relatively lower image noise with a higher iodine contrast‐to‐noise ratio (CNR).^43^, ^45^ Given their clinical utility and importance, SNRs of VMI 40 keV and VMI 70 keV are evaluated in this study.

#### 2.3.3 AUMC calculation

To assess overall spectral image quality, a noise metric termed the area under the mono‐energy curve (AUMC) is introduced. AUMC is calculated as the integral of the inverse of the mono E CRLB standard deviation estimates over a defined energy range, from 40 to 140 keV (Figure 4).

(13) $$\mathrm{AUMC}\;=\int_{E=40}^{140}\frac{1}{\sigma_{MonoE}\left(E\right)}\;dE$$

**FIGURE 4:**
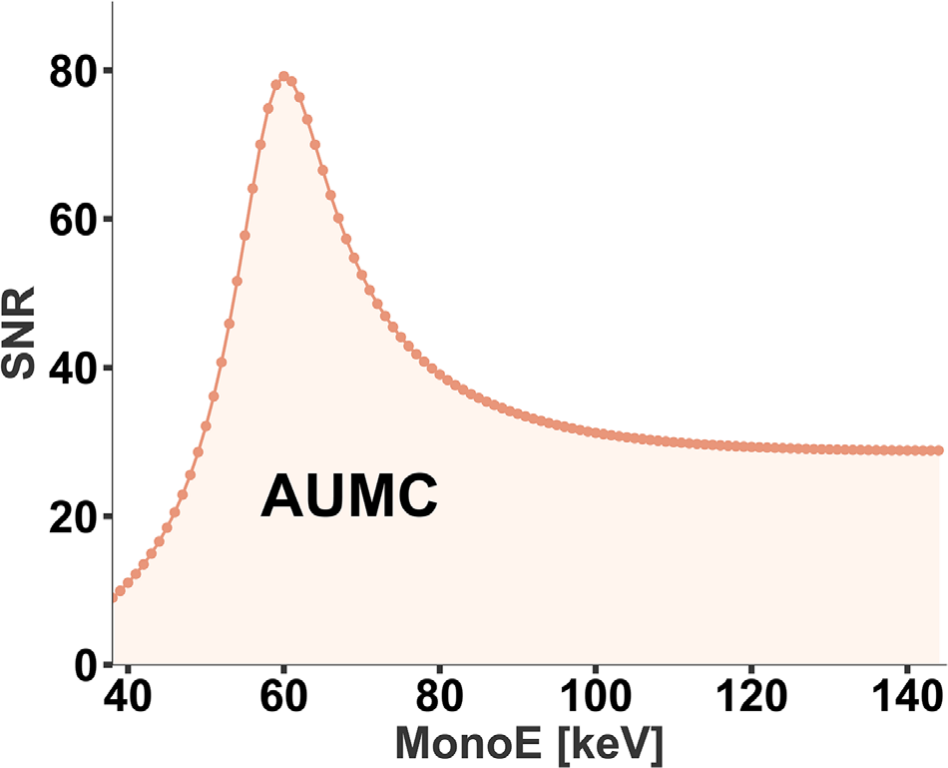
Plot depicting monoenergetic inverse noise values and the AUMC integral area for a kVp‐switching system (110/70 kVp, 100 mA, 10/90 duty cycle).

This metric provides a comprehensive assessment of spectral system performance by capturing the cumulative inverse noise across the VMI energy spectrum. The integration limits are not fixed by the method and can be adapted to the energy range most relevant to a given clinical task.

#### 2.3.4 Dose calculation

In our simulation, dose is estimated by air kerma, which measures the kinetic energy transferred from the photon beam to charged particles per unit mass. Dose evaluation was based on the total photon energy per kVp switching cycle. The simulation uses a voltage‐dependent tube emission model specifying the number of photons emitted per solid angle, unit time, and tube current to generate tube spectra $\varphi_{s}\left(V,E\right)$. The tube current itself is also voltage‐dependent and can be estimated assuming a constant filament temperature of the cathode. The voltage transition times depend on the emission current and can be estimated accordingly. With the specified tube voltages, duty cycle, and transition times, the voltage and current waveforms V(t), I(V(t)) can be estimated. Since we are interested in the dose, the effective beam filtration within the tube, additional system filtration, and especially the bowtie and K‐edge filtration, are summarized with

(14) $$\varphi_{f}\;\left(V,E\right)=\varphi_{s}\left(V,E\right)e^{-\sum_{m\in Filter}l_{m}\mu_{m}\left(E\right)}\;$$

The total photon energy D per switching cycle becomes

(15) $$D=\int_{0}^{t_{Cycle}}\int_{0}^{\infty }E\;\cdot \varphi_{f}\left(V,E\right)\;dE\;dt$$

### 2.4 Double bowtie design

The present design considers only the orthogonal projection, with the beam incident perpendicularly to the bed.

#### 2.4.1 Traditional bowtie design: only Teflon

A reference system with the traditional bowtie under reference settings (110/70 kVp, 100 mA, 10/90 duty cycle) served as a baseline. The bowtie thickness was assumed to be 0 mm at the center ray, and three conventional Teflon bowtie filters, one for each patient size, were designed to achieve a uniform non‐spectral detector SNR for cylindrical objects placed at the ISO center. The estimated patient radiation exposure profile was determined by attenuating the X‐ray beam spectrum according to the Teflon path length at each fan angle. The resulting simulated dose and performance evaluations from the Teflon bowtie filter at given fan beam angles served as reference values for evaluating the double bowtie.

#### 2.4.2 Center optimization: only K‐edge material

The double bowtie design started from the center because no Teflon was assumed there, allowing the impact of K‐edge materials to be evaluated independently. Non‐radioactive and accessible K‐edge materials, gadolinium (Gd), holmium (Ho), erbium (Er), silver (Ag), and tin (Sn), were tested at varying filter thicknesses less than 1 mm. Due to the additional attenuation introduced by K‐edge materials, tube parameters (tube current and duty cycle) were adjusted to ensure the patient dose remained consistent with the reference system. Tube current was maximally limited by X‐ray generator power (80 kW), while the duty cycle was limited by the speed of switching mechanisms and a minimum integration period width. For each K‐edge material thickness under the adjusted parameters, updated AUMC as well as $SNR_{40keV}$ and $SNR_{70keV}$ were calculated and compared to the reference value without K‐edge filtration.

Higher tube output leads to faster kV transition and less spectral overlap, which also contributes to the AUMC improvement. Since both K‐edge thickness and tube power were adjusted simultaneously, it was essential to isolate the contribution of K‐edge materials to performance improvement. Titanium (Ti), which lacks a K‐edge in this energy regime, served as a control material. Once the optimal K‐edge material and system settings were determined, Ti was tested at varying thicknesses under the same scan parameters until it produced the dose equivalent to the reference. This ensured any observed performance enhancement was attributed solely to the K‐edge effect rather than increased tube efficiency.

#### 2.4.3 Full fan‐angle optimization: combination of Teflon and K‐edge material

The thickness and system settings that provided the highest relative AUMC were selected for the full fan angle filter design.

*Flat double‐bowtie*: For the simplest configuration, a flat K‐edge material filter with uniform optimized thickness across all fan angles was used. Due to the fan beam geometry, the path length through the flat K‐edge material increased toward the edges, leading to higher attenuation. The Teflon path length was optimized to ensure the dose profile matched the reference system. After updating the filter profile, the SNR was calculated for monoenergetic images at 40 and 70 keV, along with the overall system performance metric, AUMC.

*Fully‐optimized double‐bowtie*: Based on this, the thickness of both the Teflon bowtie and the K‐edge material filter was adjusted simultaneously to achieve optimal performance across all fan angles. The objective was to maximize the AUMC value while maintaining dose consistency with the reference system. The SciPy Nelder‐Mead method in Python was used to match the dose at every fan angle.

## 3 RESULTS

For the reference system, the generated reference bowties create various dose and AUMC profiles for each patient size (Figure 5a). At the center ray, increasing K‐edge material thickness led to higher tube current and duty cycle to compensate for increased attenuation. Tube current reached the maximum allowed at 0.28 mm for Gd and Ho, and at 0.29 mm for Er. Beyond these thicknesses, tube current remained at its peak value of 727 mA, and further dose compensation was achieved by increasing the duty cycle (Figure 5b). The AUMC initially increased with filter thickness but eventually decreased as tube power became limited. The highest AUMC values were achieved at 0.47 mm for Gd and 0.43 mm for both Ho and Er. These peak values occurred at duty cycles of 0.26 for Gd and Ho, and 0.25 for Er. Importantly, the total dose remained constant and matched the reference across all tested configurations.

**FIGURE 5:**
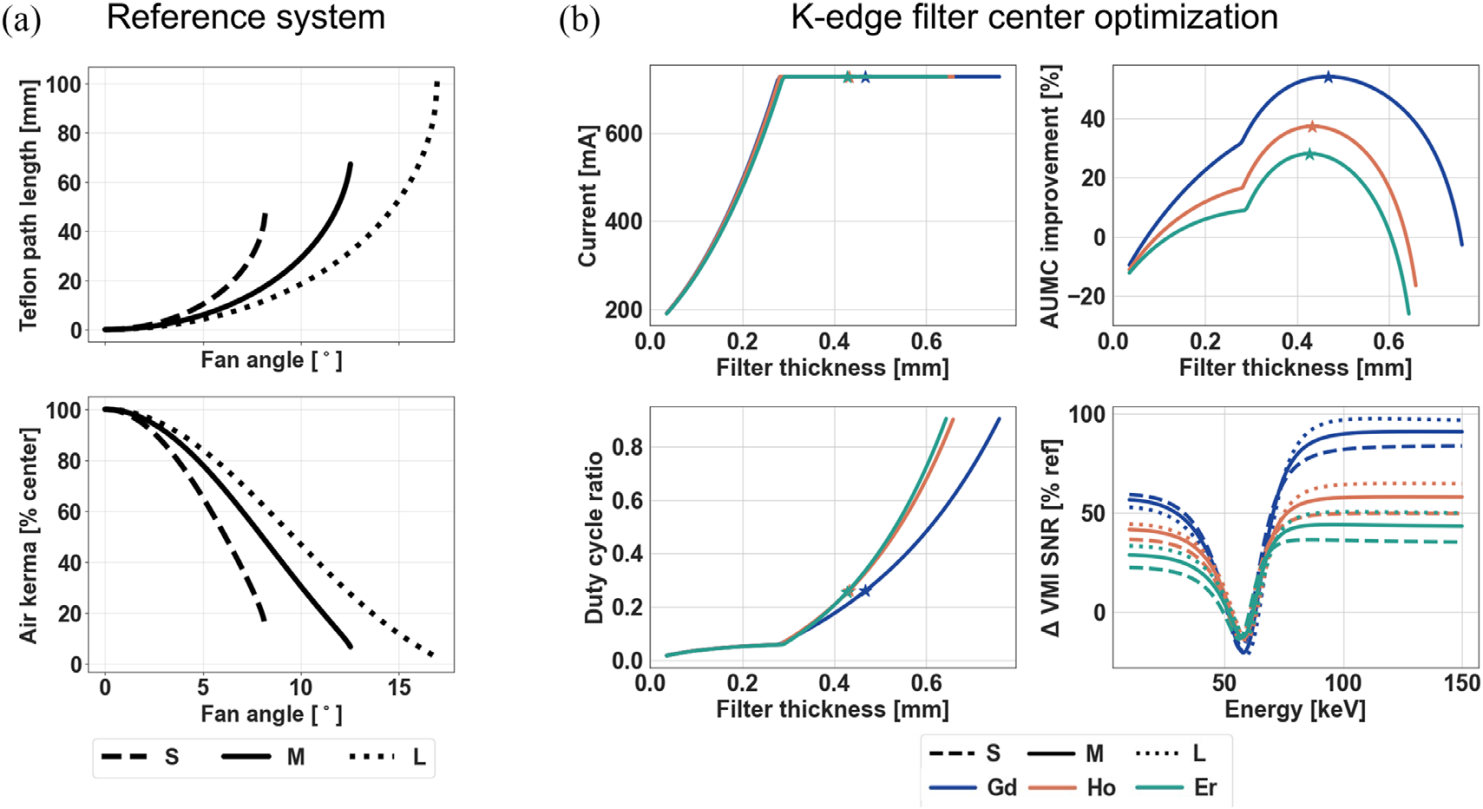
(a) Traditional Teflon bowtie profile and dose. *Top*: Teflon bowtie path length at every fan angle for three phantom sizes. *Bottom*: Phantom dose profile compared to the center for each phantom size. (b) Center filter optimization procedure. *Top Left*: Adjusted tube current to maintain the required photon fluence through the filter as its thickness increases. *Bottom left*: Duty cycle ratio for the high‐kVp tube voltage, adjusted in response to increasing filter thickness. *Top right*: AUMC values across varying filter thicknesses for three K‐edge materials, compared to the reference at the center. The filter thickness at which each material achieved its highest AUMC is marked with a star. *Bottom right*: SNR of mono energies relative to the reference system with optimal K‐edge filter thickness. Line styles represent phantom sizes, and color represents different filter materials.

For the central ray, the SNR at each VMI energy level using the optimal K‐edge filter was calculated, and the relative improvements are presented in Figure 5b. Reference SNR values were defined separately for each phantom size. Initially, the relative SNR improvement was positive but declined and dropped to below zero, reaching a minimum around 55 keV. In the energy range between 50 and 63 keV, differences in improvement between phantom sizes and materials were minimal. Beyond this range, the positive SNR improvement increased steadily with energy, eventually reaching a plateau: approximately 90% for Gd, 60% for Ho, and 42% for Er. Among the phantoms, the largest showed the greatest overall improvement, with differences of up to 7% compared to the smallest phantom.

Figure 6 shows the improvement of both K‐edge and non‐K‐edge filtered systems compared to the performance of the reference system at the center. The best AUMC was achieved using a Gd thickness of 0.467 mm for the medium and large phantoms, and 0.463 mm for the small phantom. Tube currents reached the highest possible value (727 mA) under tube power limitation for tests of all K‐edge materials. For AUMC, Gd was the best K‐edge material among all materials for all phantom sizes, having 53.8% average improvement in the three phantom sizes. Ho and Er also increased the AUMC by 37.1% and 27.9% on average. Ag and Sn performed similarly, having relatively stable AUMC improvement of around 21% for all phantom sizes. Ti, which lacks a K‐edge, reduced AUMC by roughly 6.8% at the center under its own optimized settings. For all materials, larger phantoms had greater improvement than smaller ones compared to the corresponding reference system.

**FIGURE 6:**
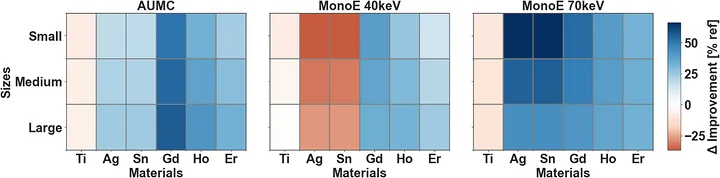
System performance under the highest AUMC conditions for different K‐edge materials. The *left* panel shows maximum AUMC values relative to the reference across phantom sizes; the *middle* and *right* panels display corresponding SNR improvements at 40 and 70 keV, respectively.

Ho and Er show comparable performance at both 40 and 70 keV, with less than 10% difference for each energy level. Ag and Sn had approximately 55% improvements in 70 keV SNR, which were the highest among all K‐edge materials, but the 40 keV SNRs were 32% worse than the reference. The suboptimal performance of Ag and Sn can be attributed to their K‐edge positions (25 and 29 keV). In contrast, Gd provided consistently strong improvements at both 40 and 70 keV, making it the most balanced performer across the spectrum.

The flat K‐edge filter double bowtie design incorporated a K‐edge filter with uniform thickness, which was determined based on center‐ray optimization. The three best‐performing materials, Gd, Ho, and Er, were selected for full fan‐angle evaluation. Due to the fan beam geometry, the effective path length through the K‐edge filter increased with fan angle. To maintain dose consistency, the Teflon bowtie was slightly modified from the reference geometry, resulting in path lengths marginally higher (less than 5%) than those in the reference system. Most of the additional attenuation introduced by the K‐edge layer was compensated by adjustments to the tube current and duty cycle.

All three K‐edge materials showed strong AUMC performance across the full fan range. Gd achieved the greatest overall improvement, with an average AUMC increase of 51.2%, followed by Ho at 36.5% and Er at 27.7% (Table 1). The maximum improvement occurred at the center, where the patient thickness was largest. As the fan angle increased, AUMC gradually decreased, with Gd showing the steepest drop (∼15%) from center to edge. Ti was tested under the same tube settings as Gd with a calibrated thickness matching Gd's attenuation profile. While the Ti filter showed a slight AUMC gain of ∼2% at the center, this advantage diminished rapidly toward the phantom edge, where the AUMC decreased to −13.8%. With the comparison between Gd and Ti, the K‐edge material showed a contribution to AUMC improvement isolated from tube current increases.

**TABLE 1:** Performance evaluation of double bowtie with flat K‐edge filter for medium phantom over all fan angles.

	Δ SNR improvement [% ref]						Δ AUMC improvement [% ref]	
	VMI 40 keV		VMI 70 keV		Best VMI			
Filter	Mean	Min / Max	Mean	Min / Max	Mean	Min / Max	Mean	Min / Max
Teflon + Ti	−65.1	−68.6 / −63.9	−18.5	−36.4 / −11.6	0.5	−9.7 / 4.3	−2.3	−13.8 / 1.9
Teflon + Gd	32.3	15.7 / 37.0	43.9	25.9 / 49.1	−3.0	−10.1 / −0.9	51.2	39.0 / 54.6
Teflon + Ho	29.2	23.7 / 30.6	36.9	27.0 / 39.8	−3.0	−8.0 / −1.5	36.5	31.2 / 38.0
Teflon + Er	20.4	18.3 / 20.9	31.6	24.4 / 33.8	−2.6	−6.8 / −1.3	27.7	24.4 / 28.7

*Note*: The best VMI change is slightly negative because the design optimizes the broadband AUMC rather than single‐energy performance at the noise minimum.

Since the flat double bowtie profile was designed, it could be used as a baseline for further optimization, where the path lengths of Teflon and K‐edge filters were optimized. For Ho, Er, and Ti, their path lengths were lower than the previous optimized value at the center, and Teflon's path length increased (Figure 7). The relative AUMC improvement showed a similar trend with a flat double bowtie. Compared with the fully optimized configurations, the flat double bowtie achieved AUMC improvements within 10% on average for Gd, Ho, and Er, demonstrating comparable performance with a simple geometry. Regardless of geometry, the Ti double bowtie consistently underperformed compared with the K‐edge‐based designs.

**FIGURE 7:**
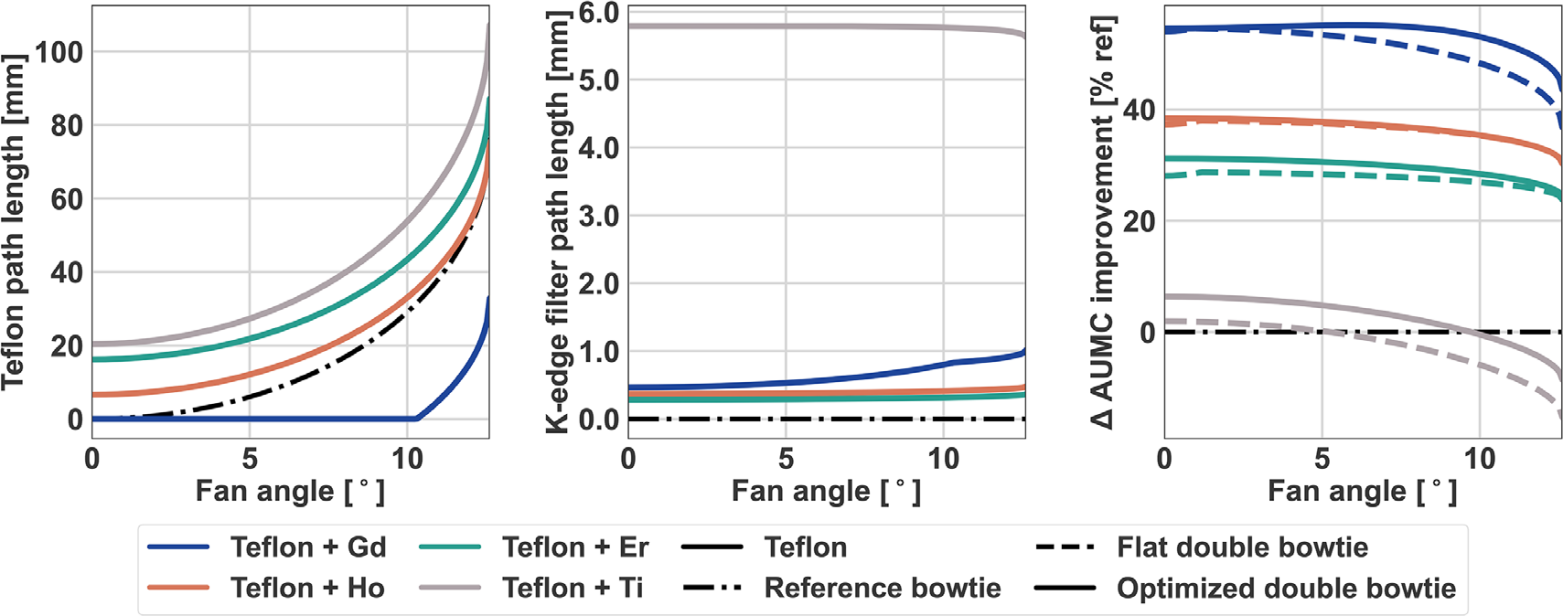
Double bowtie design with fully optimized K‐edge filter for medium phantom size. *Left*: Path length of Teflon bowtie. *Middle*: Path length of filters as the ray passes through. *Right*: The AUMC improvement is compared to the reference. The black dash‐dotted line represents the reference bowtie (no K‐edge filter). The solid lines represent the fully optimized double bowtie, and dashed lines represent the double bowtie with a flat K‐edge filter.

As Teflon + Gd was the best combination in the fully optimized double bowtie configuration, it was further optimized for three phantom sizes (small, medium, and large). Table 2 presents the percentage improvements in VMI 40 keV, VMI 70 keV, best VMI and AUMC across these phantoms relative to their respective references. For VMI 40 keV, the average SNR improvement remained consistent across sizes, with values of 46.6%, 46.3%, and 45.6% for small, medium, and large phantoms. At VMI 70 keV, the smaller phantom also had higher improvement. While the small phantom achieved the highest peak value (up to around 54%), the large phantom showed the greatest variability. Because the system was optimized for the best AUMC, the mean SNR change at each best monoenergetic energy was negative across phantom sizes, in contrast to the substantial SNR improvements observed at 40 and 70 keV. This suggests that the optimization primarily benefited AUMC rather than SNR at the best monoenergetic energies.

**TABLE 2:** Performance evaluation of Teflon + Gd fully optimized double bowtie for different phantom sizes.

	Δ SNR improvement [% ref]						Δ AUMC improvement [% ref]	
	VMI 40 keV		VMI 70 keV		Best VMI			
Phantom	Mean	Min / Max	Mean	Min / Max	Mean	Min / Max	Mean	Min / Max
Small	46.6	39.4 / 55.4	48.9	36.1 / 53.6	−5.9	−11.1 / −4.1	50.9	45.6 / 51.7
Medium	46.3	36.8 / 57.2	42.8	24.9 / 49.2	−3.5	−11.3 / −0.8	53.9	45.5 / 55.2
Large	45.6	33.9 / 58.7	34.1	8.4 / 41.8	−1.0	−13.4 / 2.8	56.0	41.3 / 58.0

For AUMC, the improvement trend rose with increasing phantom size, yielding average gains of 50.9%, 53.9%, and 56.0% for small, medium, and large phantoms, respectively. The standard deviation for the medium phantom was higher than that of the small and large phantoms. The maximum AUMC improvement occurred for the large phantom (58%).

## 4 DISCUSSION

A novel prefiltration architecture, termed the double bowtie, was developed to enhance spectral imaging performance in pediatric CT without increasing radiation dose. The design integrates a K‐edge material layer with a conventional Teflon bowtie, yielding substantial improvements in spectral signal‐to‐noise ratio across the VMI range. Among the materials evaluated, gadolinium exhibited the most consistent and robust enhancement.

In this simulation study, two filter geometries were examined for full fan‐beam optimization: a flat K‐edge filter and a thickness‐optimized configuration. The flat design achieved the highest AUMC improvement at the central ray, whereas the thickness‐optimized filter yielded more uniform SNR enhancement across all fan angles. Despite these geometric differences, their overall performance was comparable, indicating that the simpler flat K‐edge filter represents a practical and manufacturable alternative. This simplicity is advantageous for clinical translation, where fabrication efficiency is a key consideration. Furthermore, the high attenuation of K‐edge materials allows for substantial reductions in total filter thickness, conserving space within the collimation assembly.

The performance of the double bowtie depends on the interaction between the K‐edge material and the tube operating parameters. When optimized concurrently, both tube current and duty cycle rose with filter thickness, shortening the voltage transitions and extending the high‐kVp interval, which together enhanced spectral separation by minimizing intermediate‐kVp. The 110/70 pair is the result of an optimization for small objects, such as those expected in pediatric imaging, and achieves a higher AUMC than the 140/80 pair for this object size in our comparison. The reduced difference in voltages also enables faster switching and shorter duty cycles. The photons detected during the voltage transitions are assigned to one of the two IPs. The triggering of the detector is controlled by the voltage passing a given threshold, which is set to 25% of the voltage difference above the low kVp in this case. This results in a slightly different effective spectrum compared to the plateau. The simulation (used for AUMC estimation) and the material decomposition both estimate and use these effective spectra. Faster switching—that is, a steeper voltage transition—reduces the spectral difference between the ramp and plateau portions and yields improved spectral separation.

Because K‐edge filtration modifies the region of spectral overlap, the optimal filter material and energy pair depend on the underlying spectral shapes and, consequently, on the system architecture and selected tube‐voltage combination. Prior work found holmium best in dual‐layer systems and gadolinium best in kVp‐switching configurations.^34^ In our configuration, gadolinium's K‐edge at 50.2 keV falls near the upper part of the low‐kVp spectrum, so it preferentially attenuates the overlap while sparing the high‐energy photons of the high‐kVp beam, widening the effective separation and lowering CRLB noise across the VMI range. Silver and tin (K‐edges at 25 and 29 keV) act below the overlap and do not reshape it, while holmium and erbium (55.6 and 57.5 keV) sit slightly above gadolinium and reshape it less effectively. The mild SNR reduction near 50 to 60 keV reflects a redistribution of decomposition noise, with the filter improving SNR at low and high energies at the cost of a modest increase near the energy where the unfiltered system is already at its minimum. This also reflects a preference for broad spectral performance over peak performance at a single energy, which can come at the expense of more clinically important targets such as 40 keV iodine contrast.

Titanium, a non–K‐edge reference, was tested both under its own optimized tube settings and under gadolinium's settings at a matched dose. In neither case did it reproduce the K‐edge gain, isolating the K‐edge contribution from the tube‐output adjustments. Although titanium does not reproduce K‐edge–induced spectral shaping, spectral SNR is determined by the transmitted spectral shape and the resulting separation between energy channels; therefore, the comparison still isolates the effect.

Yao et al.^46^ demonstrated the feasibility of a K‐edge prefiltration design for spectral CT using simulation, reporting up to a twofold increase in spectral SNR through optimized K‐edge layering under fixed‐dose conditions. Wang et al.^47^ also showed the potential of spectral optimization using fast kV‐switching and filtration to reduce noise in material decomposition. The present work extends the concept by (1) systematically comparing multiple K‐edge materials, (2) introducing the AUMC metric to quantify spectral performance across the full energy range, and (3) evaluating manufacturable geometries—both flat and thickness‐optimized—within a full fan‐beam configuration. Additional approaches to enable spectral CT through K‐edge filtration have included the use of commercially available split‐beam systems^9^, ^48^ and experimental prototypes incorporating dynamically controlled or spatially varying multi‐material filters.^14^, ^49^, ^50^ However, such designs rely on complex hardware architectures that are less compatible with clinical implementation in pediatric imaging, where simplicity, stability, and manufacturability are critical.

Several limitations should be acknowledged. Although optimized bowtie profiles were generated for three pediatric phantom sizes, clinical implementation must accommodate a broader range of patient anatomies and potential size mismatches. Off‐center patient positioning, which frequently occurs in practice, may also affect dose uniformity and image quality.^51^ Additionally, the increased tube current and duty cycle required for optimal operation demand higher tube power and energy consumption, potentially raising concerns about system power limits and tube longevity. The present design is therefore most applicable to pediatric imaging, where lower tube power settings are standard, and may require modification for adult imaging protocols. Because the design is optimized for the 110/70 protocol, other spectral pairs would require re‐optimization, and as the double bowtie is an add‐on rather than a replacement, non‐spectral and alternative protocols retain the standard bowtie.

As a projection‐domain study, the framework does not model scatter, the heel effect, or image‐domain beam‐hardening artifacts. The spectral gains are therefore best interpreted as a system‐level comparison under matched conditions rather than a prediction of reconstructed image quality.

Finally, the radiation dose was estimated using air kerma as a surrogate. Air kerma and absorbed photon energy were shown to exhibit similar spatial distributions.^52^ We further confirmed this by repeating the full optimization with absorbed dose, which produced the same material ranking and optimal design with only minor differences in absolute values. Both estimates, however, are computed in a 2D fan‐beam geometry and represent energy along the in‐plane primary beam. They do not capture the longitudinal (*z*‐axis) dose distribution, scatter that redistributes dose radially and along the axis, or the depth‐dependent falloff that full 3D modeling would provide. Clinically relevant 3D indices such as CTDI_vol_, SSDE, or Monte Carlo calculations, together with full 3D modeling of dose and scatter, would offer a more complete characterization of patient exposure at the cost of additional computation. Since the present study aims to compare configurations rather than report absolute exposure values, the matched‐dose 2D framework used here is sufficient for this purpose. Likewise, AUMC and the CRLB analysis characterize spectral performance and decomposition precision rather than a specific clinical task, though the framework extends naturally to a water/iodine basis for future task‐based evaluation.

Future work will include advanced simulations and benchtop experiments to evaluate the impact of the double‐bowtie design on image quality and its potential for integration into clinical spectral CT systems.

## 5 CONCLUSION

In this study, we present a bowtie filter design integrated with spectral CT system modulation to enhance spectral performance while maintaining low radiation dose levels suitable for pediatric imaging. The design combines a conventional Teflon bowtie with a K‐edge material layer optimized across discrete fan angles to improve spectral separation. By incorporating a strategically selected K‐edge component, the filter selectively shapes the X‐ray spectrum, thereby increasing energy separation and enhancing spectral SNR. Although the current implementation is demonstrated within a kVp‐switching CT system, the double‐bowtie concept is broadly applicable to other spectral CT technologies and does not require dedicated pediatric hardware. As a pediatric‐specific adaptation, this design enables children to benefit fully from the diagnostic advantages of spectral imaging.

## CONFLICT OF INTEREST STATEMENT

Peter B. Noël has received a hardware grant from Philips Healthcare. Peter B. Noël receives research grant funding from Philips Healthcare. Amy E. Perkins, Kevin M. Brown, Yannan Jin, and Ravindra M. Manjeshwar are employees of Philips Healthcare. Heiner Daerr and Thomas Koehler are employees of Philips Innovative Technologies. The other authors have no relevant conflicts of interest to disclose.

## Data Availability

The data that support the findings of this study are available from the corresponding author upon reasonable request.
